# Drilling Force Characterization during Inconel 718 Drilling: A Comparative Study between Numerical and Analytical Approaches

**DOI:** 10.3390/ma14174820

**Published:** 2021-08-25

**Authors:** Salman Pervaiz, Wael A. Samad

**Affiliations:** Department of Mechanical and Industrial Engineering, Rochester Institute of Technology—Dubai Campus, Dubai P.O. Box 341055, United Arab Emirates; wascad@rit.edu

**Keywords:** Inconel, drilling force, FEA, modeling material removal

## Abstract

In drilling operations, cutting forces are one of the major machinability indicators that contribute significantly towards the deviations in workpiece form and surface tolerances. The ability to predict and model forces in such operations is also essential as the cutting forces play a key role in the induced vibrations and wear on the cutting tool. More specifically, Inconel 718—a nickel-based super alloy that is primarily used in the construction of jet engine turbines, nuclear reactors, submarines and steam power plants—is the workpiece material used in the work presented here. In this study, both mechanistic and finite element models were developed. The finite element model uses the power law that has the ability to incorporate strain hardening, strain rate sensitivity as well as thermal softening phenomena in the workpiece materials. The model was validated by comparing it against an analytical mechanistic model that considers the three drilling stages associated with the drilling operation on a workpiece containing a pilot hole. Both analytical and FE models were compared and the results were found to be in good agreement at different cutting speeds and feed rates. Comparing the average forces of stage II and stage III of the two approaches revealed a discrepancy of 11% and 7% at most. This study can be utilized in various virtual drilling scenarios to investigate the influence of different process and geometric parameters.

## Highlights

- A mechanistic model and FEA assisted cutting simulation model were developed and compared for the drilling of Inconel 718.
- Convex profile of the drilling force profile at entry versus time was observed in both models.
- Temperature profile was correlated with the higher feed rate as well as higher cutting speeds.
- Discrepancies were seen at higher cutting speeds were associated with thermal softening phenomena.

## 1. Introduction

Metal cutting operations such as turning, milling and drilling are widely used in manufacturing for the purpose of reducing a variety of mechanical components and structures. Drilling, a hole producing process, is especially important because it accounts for a large portion of overall machining operations. Additionally, drilling problems can result in costly production waste because many drilling operations are often among the final steps in fabricating a part.

Before diving into analytical and finite element formulations of drilling, it is worth noting that the very first approach in the drilling community was based on experimental measurements, commonly referred to as phenomenological modeling [1]. This approach provides a feed force and torque depending on the cutting speed and feed rate for a specific cutting tool and workpiece material. While this approach is advantageous in conventional drilling scenarios and is able to provide accurate predictions [2], such phenomenological models suffer from poor extensibility [3,4]. Additionally, any design iteration or manufacturing modification applied to the drilling tool piece would result in months-delay before experimental validation can be possible [5]. As a consequence, the development of analytical and finite element models to accurately predict forces for different drilling scenarios has been a focus in the manufacturing industry. 

In analytical modelling, the majority of research in literature uses the discretization approach of cutting edges as their fundamentals, such as the ones presented in [6,7,8,9,10]. The feed force and torque are calculated by summing the local forces along the cutting edge of the tool piece. The local forces, which depend on the normal rake angle $\alpha_{n}$, inclination angle i, cutting velocity $V_{c}$, uncut chip thickness $t_{c}$, and the friction coefficient, are predicted using different methods. For instance, a common way is to rely on experimental data related to the drilling of a particular workpiece using a specific drill. Such an approach is obviously tool and work piece specific and is thus difficult to generalize since different tool geometries would lead to vast differences in cutting angles and velocities along the cutting edges. An alternate way to modeling the drilling force profiles relies on using generic cutting tests in order to identify the analytical flow stress model of the work piece. Examples of such an approach include the Oxley model which is used in [9,10]. While those have been fairly successful, they still rely on parameters that are confined to specific work materials. Further advancements in analytical modeling include the findings presented in [11], where the link between the cutting forces and its distribution along the cutting edges to the cutting parameters the drill geometry and the cooling conditions are established. Finally, it is worth noting that all models consider a constant friction when it has not been quantified by tribological experiments [5]. This is important for capturing cutting speed effects as the friction coefficient can vary from with decreasing cutting speeds. Such dependency is consistent with the variation of the cutting speed along the edge of a drill for steel [12].

Fernandes et al. [13] developed a 3D finite element assisted drilling simulation model based on the rigid foam material similar to human bone. The model was built to reveal the load intensity distribution during the drilling process. The study revealed that as the drill penetrates and hole depth increases the stress level also increases as well. The drilling model provided a means to study different process and geometric parameters without incorporating the actual experimentation. Giasin et al. [14] investigated the drilling performance of Al2024-T3 aerospace alloy using TiAlN-coated carbide. The hole quality was inspected using chip formation and burr, surface finish, hole diameter measurement and microhardness. The study revealed a stronger influence of process parameters on the drilling performance. Doomra et al. [15] performed a numerical study towards the drilling performance of Al1100/10% SiC metal matrix composite (MMC). Thrust force was simulated using a finite element drilling simulation model and it was found in good agreement with the experimental data. It has been found that higher feed rate and cutting speed increases the thrust force as well. Ucun, [16] conducted a numerical study to simulate drilling process on Al7075-T6 alloy using the twist drill and three flute drill. The study used cutting forces, torque and stress on the tool using the FE numerical model. The study revealed better performance of twist drill as compared with the three-flute drill with lower forces, torque and stress. Nagaraj et al. [17] performed a numerical study to investigate the drilling performance of Nimonic C-263. The simulations were performed in reference to a Taguchi L27 orthogonal array-based experimental design. The study revealed good agreement between experimental and numerical results. Abdelhafeez et al. [18] developed a finite element model based on coupled Eulerian Lagrangian (CEL) approach. The study considered Ti64 and AA-2024 as workpiece materials. The thrust forces and torque were found under 20% error range with respect to the experiments. 

This current work is performed using the power law method with detailed steps using the stress strain data of Inconel 718. The coefficients for Inconel 718 barely exist in the available metal cutting literature. It is also very rare to find both mechanistic and finite element (FE) models together for the Inconel 718. The finite element analysis (FEA) assisted numerical model also incorporates a pilot hole in the current study making it unique as compared to other studies available in the literature. The presence of the pilot hole enabled us to capture the cutting force signature and cutting action of the chisel edge and cutting lip during the drilling operation.

## 2. Analytical Modelling Setup

The analytical model employed in the work presented here relies heavily on the derivations in the literature [8]. The major components of this analytical mechanistic approach are highlighted and reiterated in this section. Through the use of conservation of energy methods, whereby the chip is held in equilibrium by the resultant machining force, the cutting forces acting on the tool are proportional to the uncut chip area [19]. This area is the projected area of the shear plane measured in a plane normal to the cutting velocity. The specific cutting pressure is dependent on the cutting conditions and the tool geometry. In this manuscript, a conventional conical point drill is used, consistent with the equations developed in the literature [8]. The main geometric parameters of the drill are listed in Table 1 below.

**Figure 1:**
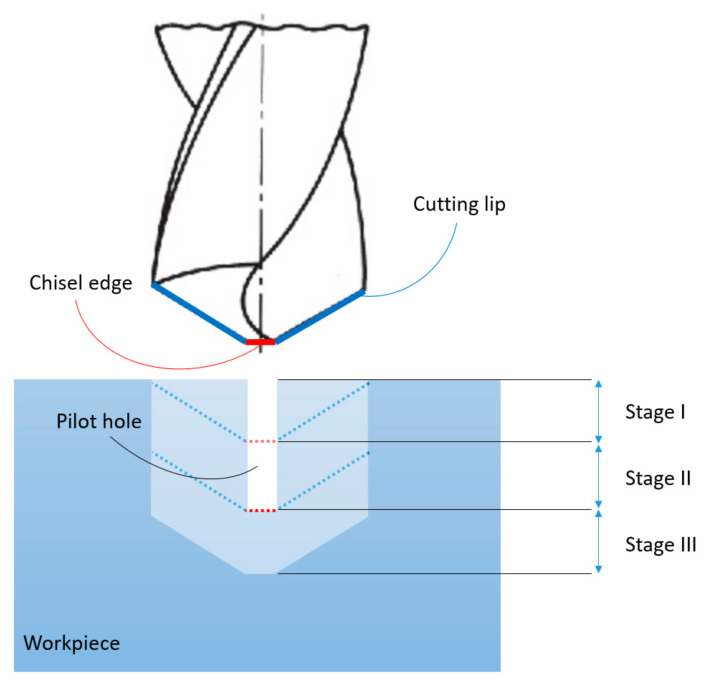
Schematic illustration of the drilling operation highlighting the three drilling stages.

The drilling operation for the case where a pilot hole is present in the workpiece can be divided into three separate stages as shown in the annotated schematic diagram of Figure 1. Stage I, often referred to as the entry stage, where the cutting lips of the drill are gradually engaged in the drilling operation. Stage II, where the entire cutting lips are fully engaged upon covering the perpendicular distance often referred to as the tip. And finally, stage III, where the chisel edge is engaged in the material removal upon the tool traveling the pilot hole distance, $d_{p}$.

Models that account for the unique nature of the cutting process are formulated and adopted from [20] and listed herein below. During entry, stage I, the force profile at is evaluated as the cumulative sum of forces from the pilot hole radius, $r_{p}$, to the outer radius, $r\left(t\right)$, of the drill that is engaged in cutting as shown in Equation (1) below:

(1) $$F_{cl}\left(t\right)=\int_{\frac{r_{p}}{R}}^{\frac{r\left(t\right)}{R}}2K_{n}\left(\rho \right)\frac{f_{rev}}{2}sin\;sin\;k\;\;cos\;cos\;i\left(\rho \right)\;\;Rd\rho$$

From which, the closed form solution for the force can be obtained as a function of the cutting time of stage I as shown in Equation (2):

(2) $$F_{cl}\left(t\right)=\frac{C_{2}Rf_{rev}\;sin\;sin\;k\;\;}{\left(C_{1}+1\right)}\left\{{\left(\frac{r\left(t\right)}{R}\right)}^{C_{1}+1}-{\left(\frac{r_{p}}{R}\right)}^{C_{1}+1}\right\}-\frac{C_{2}f_{rev}w^{2}k\;\;}{2R\left(C_{1}-1\right)}\left\{{\left(\frac{r\left(t\right)}{R}\right)}^{C_{1}-1}-{\left(\frac{r_{p}}{R}\right)}^{C_{1}-1}\right\}$$

where $r\left(t\right)$ can be determined from the drill feed rate, $f_{s}$, using Equation (3) shown below:

(3) $$r\left(t\right)=\sqrt{r_{p}^{2}+2tf_{s}\;tan\;tan\;k\;\;\sqrt{r_{p}^{2}-{\left(\frac{w}{2}\right)}^{2}}+\left(tf_{s}\;tan\;tan\;k\right){}^{2}}$$

The constants $C_{1}$ and $C_{2}$ were determined by fitting multiple FEA force entry data at different feed rates and spindle speeds, described in Section 3, to the force equation of Equation (2). With the analytical formulation of the force due to the cutting lips engagement determined, the next step is accounting for the chisel edge upon its engagement in stage III. The model used here relies on the findings in [21], where it was shown that for drilling metals, and in a small region around the center of the chisel edge, the tool extrudes the material as opposed to cutting. This region is often referred to as the indentation zone. The force due to the indentation zone is given by Equation (4) below:

(4) $$F_{ind}=\frac{4\tau_{y}\left(1+\epsilon \right)f_{rev}R_{a}\;sin\;sin\;\alpha_{n}\;\;\;}{cos\;cos\;\alpha_{n}\;-sin\;sin\;\left(\alpha_{n}-\epsilon \right)\;\;}$$

where, the chisel edge normal rake angle $\alpha_{n}$ is determined from the point angle, k, and the chisel edge angle, ψ, using Equation (5) below:

(5) $$\alpha_{n}=-\left(\;\left(tan\;tan\;k\;cos\;cos\;\left(\pi -\psi \right)\;\;\right)\;\;\right)$$

whereas, the term ϵ in Equation (4) is determined from the boundary conditions of the problem through solving the nonlinear equation of Equation (6) using MATLAB’s *fzero* nonlinear function solver:

(6) $$\epsilon +\left[tan\;tan\;\left(\frac{\pi }{4}-\frac{\epsilon }{2}\right)\;\right]-2\alpha_{n}=0$$

Finally, and for the case of drilling metallic alloys, the specific cutting pressures was integrated from the radius of the indentation zone, $R_{a}$, to the length of the chisel edge. $R_{a}$ here was determined from [20] using a geometric analysis of the transition point. It is worth noting that other approximations can also be used such as the one described in literature [22]:

(7) $$R_{a}=\frac{f_{rev}}{2\;tan\;tan\;\left(90-k\right)\;\;}$$

**Figure 2:**
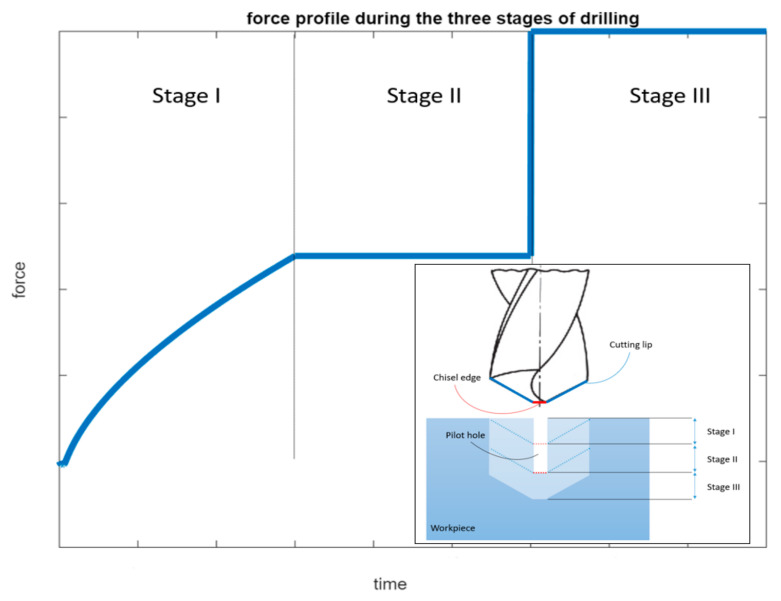
Thrust force profile during the three drilling stages.

With that, the force profile is now available from the drill bit entry and until the entire drill, cutting lips and chisel edge, are engaged in cutting as shown in the representative plot of Figure 2 below. 

Not that the convex profile of the force at entry seen in Figure 2 is consistent with experimental trends such as the ones portrayed in literature [8]. 

## 3. Finite Element Modeling Setup

The FEA model presented in this section was developed using the computer-aided engineering software AdvantEdge (Third Wave Systems, Minneapolis, MN, United States) which specializes in modeling material removal processes. The workpiece was meshed with minimum element size of 0.02 and maximum size of 0.1 for nodes. At the cutting-edge adaptive meshing was activated with respect to certain tolerance values to attain high mesh density [23]. The mesh refinement factor and the coarsening factor have been set to 2 and 6, respectively. As depicted in Equation (8), advanced power law was used to mimic the behavior of Inconel 718 workpiece material [24]. The workpiece behavior is composed of the three important phenomena namely strain hardening, strain rate sensitivity and thermal softening. The power law integrates strain hardening function as *g* ($\varepsilon^{p}$), thermal softening function as *θ* (*T*) and strain rate sensitivity function is given by *τ* ($\dot{\varepsilon }$):

(8) $$\sigma \;(\varepsilon^{p},\;T,\;\dot{\varepsilon })=g\;(\varepsilon^{p})\;\theta (T)\;\tau \;(\dot{\varepsilon })$$

**Figure 3:**
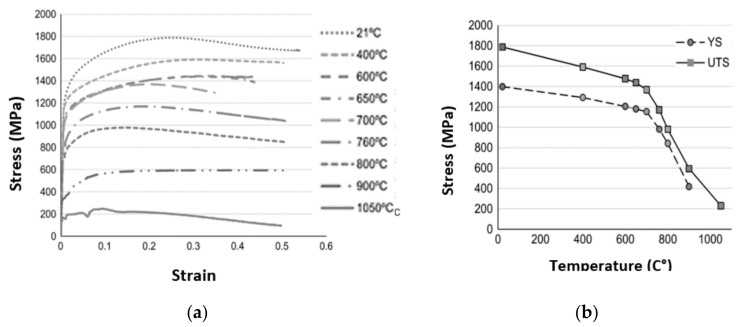
Flow stress behavior of Inconel 718 (**a**) Testing at various temperature at strain rate of 1 s^−1^ (**b**) Thermal softening behavior (obtained from [25] with kind permission from Elsevier).

**Figure 4:**
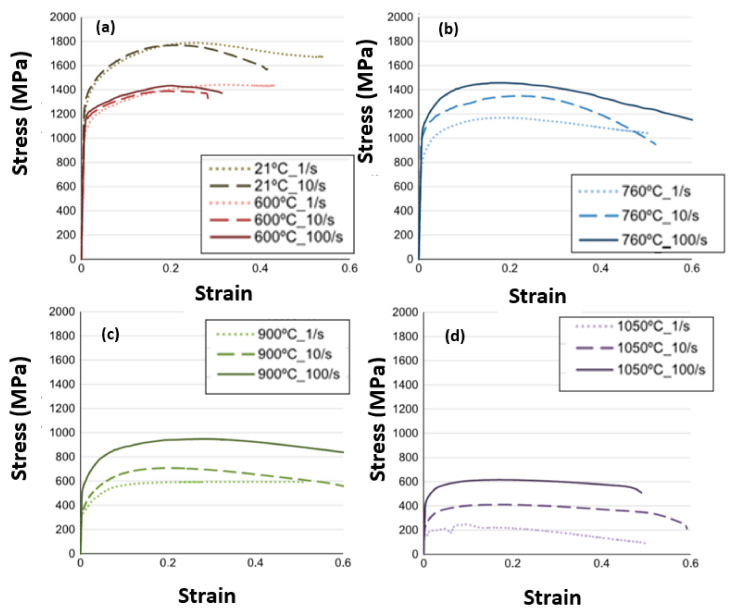
Flow stress behavior of Inconel 718, Temperature influence with respect to the various strain rates (**a**) Temperature of 21 °C and 600 °C; (**b**) Temperature of 760 °C; (**c**) Temperature of 900 °C and (**d**) Temperature of 1050 °C (obtained from [25] with kind permission from Elsevier).

The representation of strain hardening function is evaluated in Equation (9). As for strain hardening, stress-strain data of a workpiece material was examined and curve fitted to attain the governing parameters, $\varepsilon_{o}^{p}$ and *n*. Strain rate sensitivity function is modelled using Equation (10) [24]. To form precise chip shape and cutting force behavior, it is important that the model is capable of incorporating thermal softening behavior as shown in Equations (11) and (12). Thermal softening function was introduced in the power law via fifth order polynomial equation as described in Equation (11) [24]. The values of initial yield stress can be obtained from stress-strain data of uniaxial compression or tensile tests of the Inconel workpiece material at hand [25]. More specifically, experimental data of Figure 3 and Figure 4 were utilized from the work presented in literature [25] to obtain the parameters related to the hardening, thermal softening and strain rate sensitivity:

(9) $$g(\varepsilon^{p})=\sigma o\;{\left[1+\frac{\varepsilon^{p}}{\varepsilon^{p}o}\right]}^{\frac{1}{n}}\;\;\mathrm{if}\;\;\varepsilon^{p}\;\;<\;\;\varepsilon_{cut}^{p}\;$$

(10) $$\tau (\dot{\varepsilon })=\sigma o\;{\left[1+\frac{\dot{\varepsilon }}{\dot{\varepsilon }o}\right]}^{\frac{1}{m1}}\;$$

*θ(T) = c_0_ + c_1_T^1^ + c_2_T^2^ + c_3_T^3^ + c_4_T^4^ + c_5_T^5^*  if *T < T_cut_* (11)

(12) $$\theta (T)=\theta (T_{cut})(1\;-\;\frac{T-T_{cut}}{T_{m}-T_{cut}})\;\;\mathrm{if}\;T\;>\;T_{cut}$$

**Figure 5:**
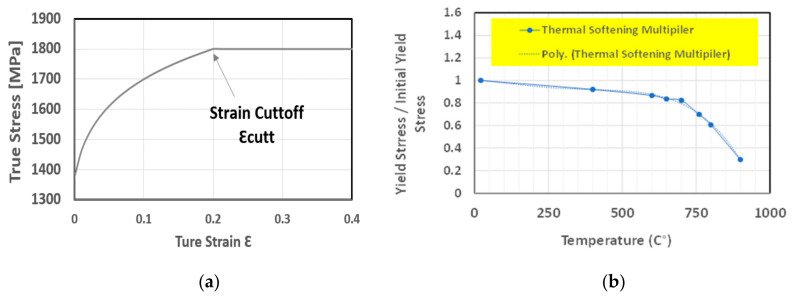
Inconel 718 Parameters for finite element model (**a**) Strain hardening behavior (**b**) Thermal softening behavior.

The AdvantEdge software manual and literature [26,27] were consulted in obtaining all the required parameters for the Inconel 718 material model. The fitted experimental stress-strain data of [25] for the determination of the strain hardening and the thermal softening behavior are shown in Figure 5 below. Also, Table 2 reveals the values of the parameters used in the equations. 

Furthermore, the AdvantEdge software uses the sliding friction model where the sliding force is proportional to the normal loading as shown in Equation [13]:

*F_s_ = μ σ_n_* (13)

Chip shape is incorporated in parallel with how damage is simulated. The AdvantEdge software simulates damage in the workpiece material by using a damage function *D* as shown in Equation (14). The fracture strain was represented using the temperature dependent model of Equation (15) [24]. In the work presented in this manuscript, the six parameters were fitted to polynomial function using the Johnson Cook damage model [28,29]. The resulting values of the parameters used are tabulated in Table 3:

(14) $$D=\overset{}{\underset{i}{\sum }}\frac{\Delta \varepsilon_{i}^{p}}{\varepsilon_{f_{i}}^{p}}$$

(15) $$\varepsilon_{fo}^{p}=d_{0}+d_{1}T^{1}+d_{2}T^{2}+d_{3}T^{3}+d_{4}T^{4}+d_{5}T^{5}$$

**Figure 7:**
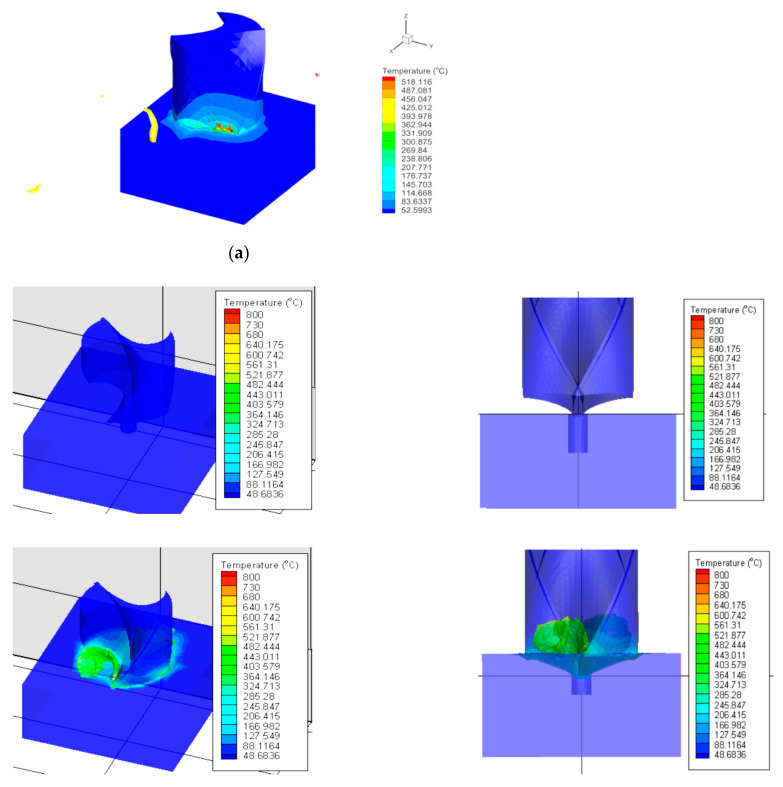

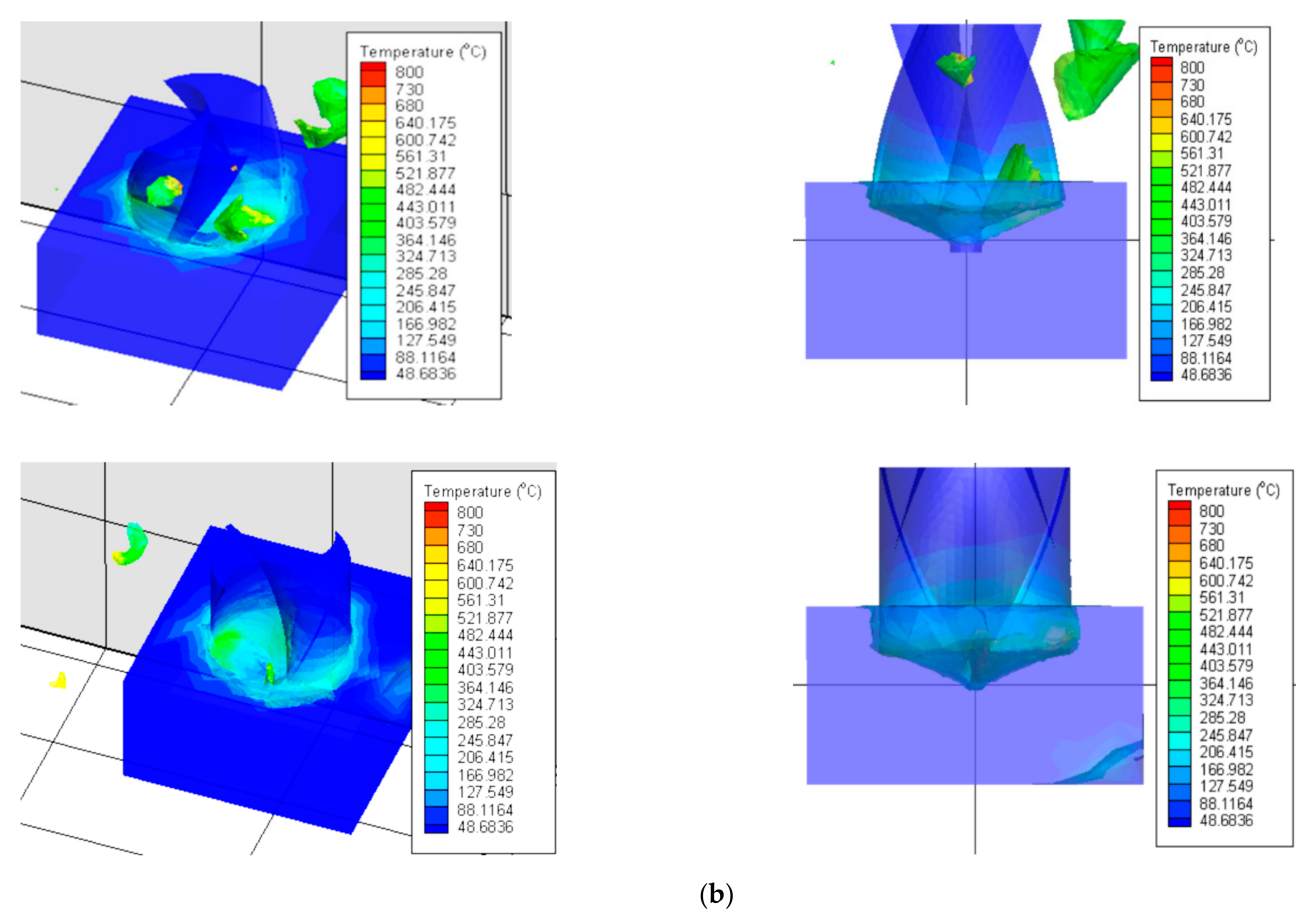
(**a**) Sample FE simulation showing chip formation for feed rate 0.375 mm/rev and 1000 spindle rpm (**b**) FE simulation (feed rate 0.75 mm/rev and 3000 spindle rpm) showing phases of starting, only cutting lips engagement with pilot hole and engagement of both cutting lip and chisel edge.

Finally, the variation of thermal conductivity and specific heat capacity with temperature for Inconel 718 were obtained from Figure 6 of [30]. A sample result of the FEA model is shown in Figure 7a, where chip formation is visible in the contour plot of the temperature distribution at a feed rate 0.375 mm/rev and 1000 spindle rpm. Figure 7b shows FE simulation (feed rate 0.75 mm/rev and 3000 spindle rpm) with the phases of starting, only cutting lips engagement with the pilot hole and engagement of both cutting lip and chisel edge.

The study modeled the cutter as a rigid material with minimum element edge length as 0.06 mm. The Workpiece was also modeled with the maximum and minimum element edge length of 1 mm and 0.07 mm. Both tool and workpiece were meshed into 40,203 elements using four-node tetrahedral elements in total. Since the Lagrangian formulation involves nodal movement with the material deformation, mesh distortions can happen in the finite element model. Mesh distortions can induce problems such as low accuracy, lower convergence rate, critical time steps, element failure and volumetric locking. To avoid the problems with element distortion, adaptive remeshing was involved in the model. As per some previous studies [31,32], the mesh refine factor and mesh coarsening factors were set to 2 and 5, respectively. 

Table 4 below shows all six simulation scenarios performed in the work presented here. Those included two feed rates with three spindle speeds for each. 

## 4. Results and Discussion

**Figure 8:**
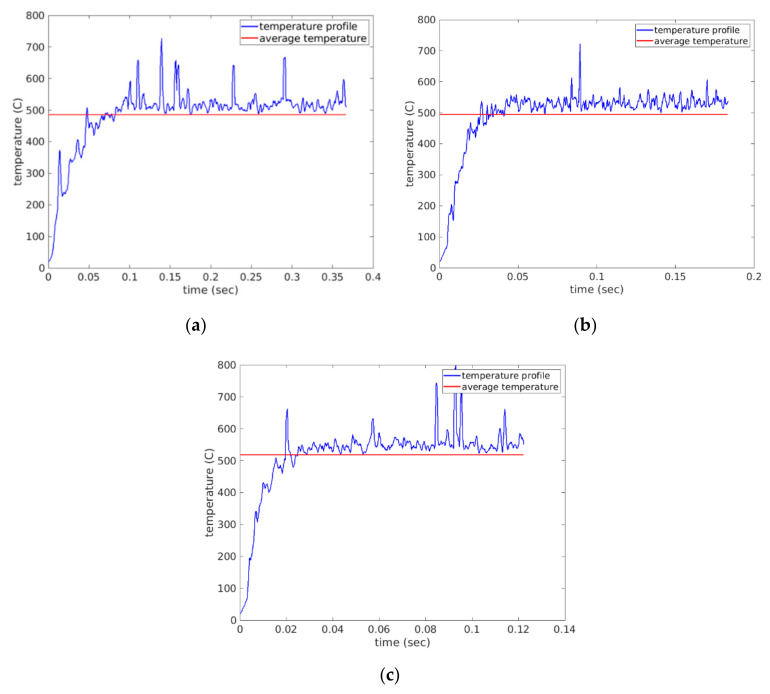
Cutting temperature profiles observed under simulations (**a**) scenario 1 of feed rate 0.75 mm/rev and 1000 spindle rpm (**b**) scenario 2 of feed rate 0.75 mm/rev and 2000 spindle rpm and (**c**) scenario 3 of feed rate 0.75 mm/rev and 3000 spindle rpm.

**Figure 9:**
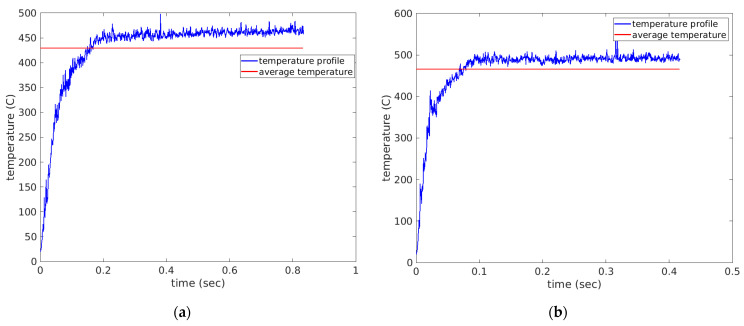

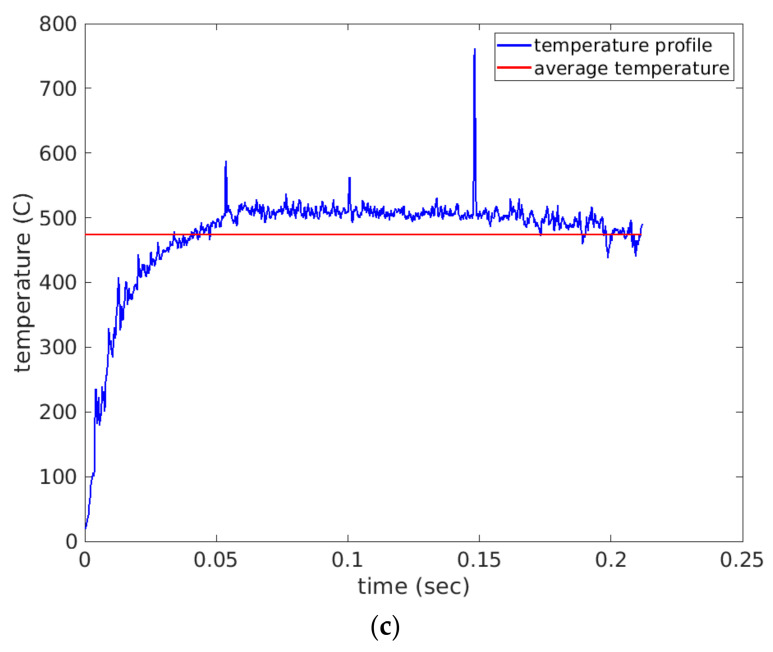
Cutting temperature profiles observed under simulations (**a**) scenario 4 of feed rate 0.375 mm/rev and 1000 spindle rpm (**b**) scenario5 of feed rate 0.375 mm/rev and 2000 spindle rpm and (**c**) scenario 6 of feed rate 0.375 mm/rev and 3000 spindle rpm.

Cutting temperature is deemed one of most important factors that has a controlling influence on the tool life and related wear mechanisms. Cutting temperature during the drilling process is also a strong function of machining parameters, workpiece and cutting tool materials and most importantly cutting environments [33]. The measurement of cutting temperature is a very demanding task since the cutting edge is always hidden with the material being cut. Thus, finite element analyses are often utilized to predict the temperature profiles during the drilling operation. Figure 8a–c shows the temperature profiles throughout the drilling operation for scenarios 1–3 respectively, while Figure 9a–c shows the temperature profiles throughout the drilling operation for scenarios 4–6, respectively. All plots were constructed in MATLAB (The MathWorks, USA) after exporting the FEA data from AdvantEdge.

**Figure 10:**
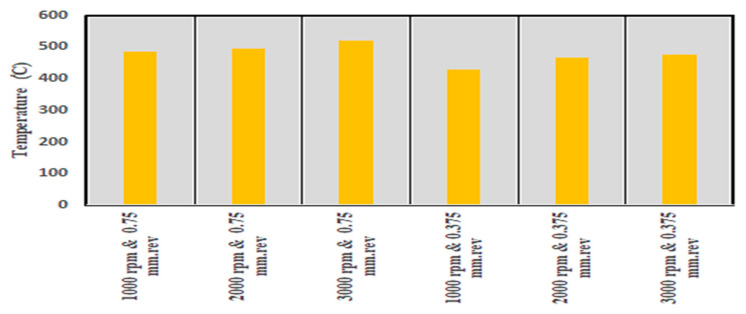
Average cutting temperature values observed for the six simulations of Table 4.

The average temperature of each scenario in Table 4 was calculated and used for further processing. The average temperature values were plotted together in the bar chart of Figure 10. It was observed that the cutting scenarios with the higher feed rate of 0.75 mm/rev provided a higher average cutting temperature than the lower feed level scenarios. This can be associated with the higher chip load at higher feed rate [8]. Higher chip load can form higher chip root temperature at the tool tip under the cutting edge. Another trend that was observed was that increasing the cutting speed increased cutting temperature as well. This is associated with the fact that increasing cutting speed increases friction at the cutting zone, thus resulting in higher cutting temperatures [30,33,34].

Cutting forces are of intricate nature in the drilling operation due to the complications involved in the drill geometry. In order to understand the complicated nature of cutting force in drilling, it is important to analyze the workpiece engagement with the chisel edge and cutting lips. The workpiece engagement with respect to the chisel edge involves indentation only, whereas the cutting lips perform the machining operation and contribute significantly towards the generation of cutting forces. The workpiece material being cut moves through the cutting lip and slides up towards the hollow helical path of the drill to exit the drilled hole. 

**Figure 11:**
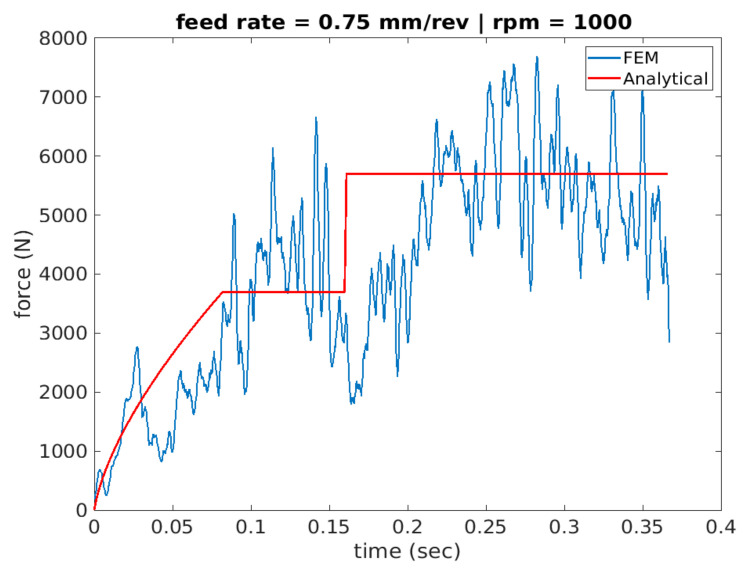
Thrust force profile for scenario 1 of feed rate 0.75 mm/rev and 1000 spindle rpm (analytical vs. FEA).

**Figure 12:**
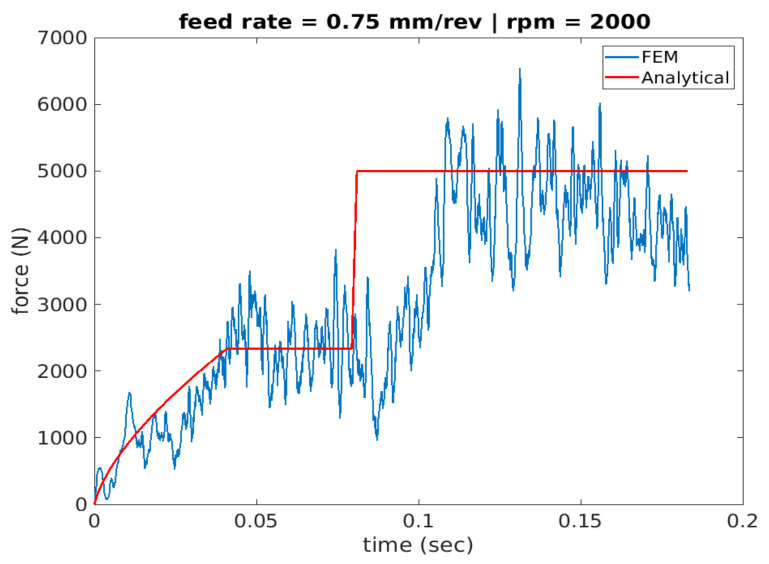
Force profile for scenario 2 of feed rate 0.75 mm/rev and 2000 spindle rpm (analytical vs. FEA).

**Figure 13:**
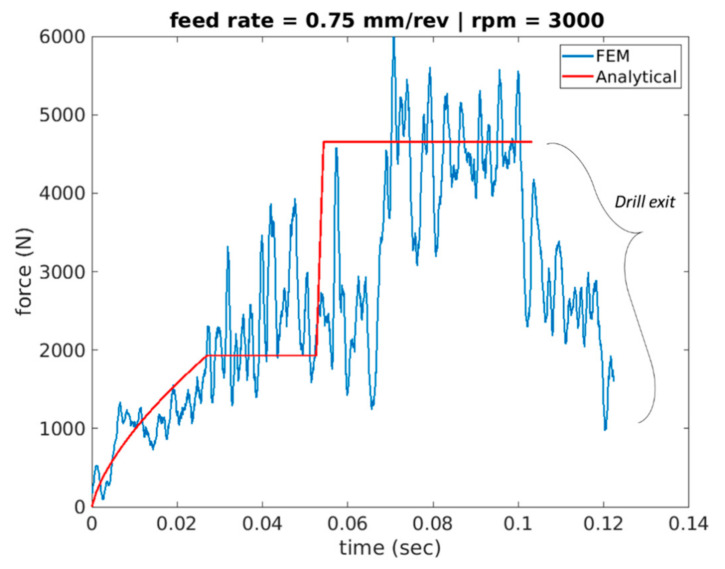
Force profile for scenario 3 of feed rate 0.75 mm/rev and 3000 spindle rpm (analytical vs. FEA).

Figure 11, Figure 12 and Figure 13 show cutting force profiles for the cutting speeds of 1000, 2000 and 3000 rpm, respectively, at constant feed of 0.75 mm/rev using both finite element analysis predictions and analytical model results. Schematic of the analytical cutting force profile is depicted in Figure 2 showing the three stages associated with the drilling operation of a workpiece geometry containing a pilot hole. It was observed that stage I consists of a ramping increase of the cutting force that depicts only the workpiece engagement of cutting lip but chip load is increasing as the drill moves inward. Increase in the chip load is linked with the increase in the cutting volume.

The second stage, stage II, is where the engagement of only cutting lip with the workpiece material was captured. In order to make sure that chisel edge cutting is not involved, workpiece material was developed using the pilot hole with diameter similar to the length of chisel edge. The second engagement appears like a step function after this initial ramping, and it is due to having the same chip load. Finally, stage III appears in the cutting force profile as another step function that has both workpiece-chisel edge and workpiece–cutting lip engagements at the same time upon the tool travelling the vertical distance, $d_{p}$, associated with the depth of the pilot hole.

The average thrust forces were calculated from both analytical and FEA approaches and the relative percentage error was calculated using the Equation (16) as shown below.

(16) $$\mathrm{Relative}\;\mathrm{Error}\;\%=\frac{\left|\;\mathrm{Analtical}\;\mathrm{Result}-\mathrm{FEM}\;\mathrm{Simulated}\;\mathrm{Result}\right|}{\mathrm{Analytical}\;\mathrm{Result}}\;\times 100$$

**Figure 14:**
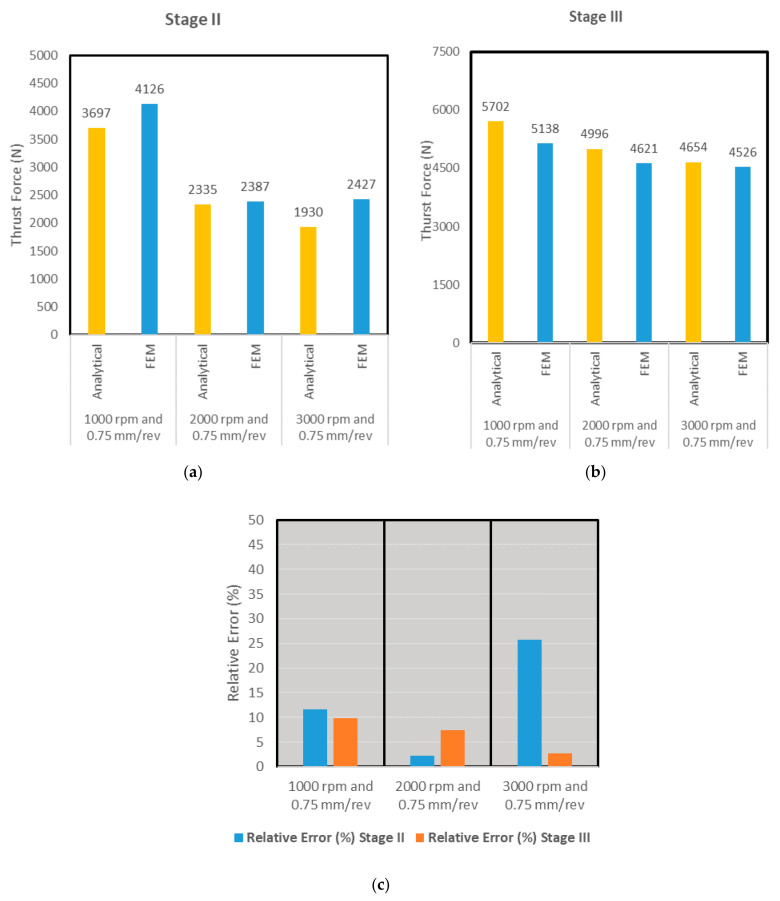
(**a**) Stage II: Average thrust forces in analytical and FEA approaches (**b**) Stage III: Average thrust forces in analytical and FEA approaches (**c**) Relative error % in both approaches.

**Figure 15:**
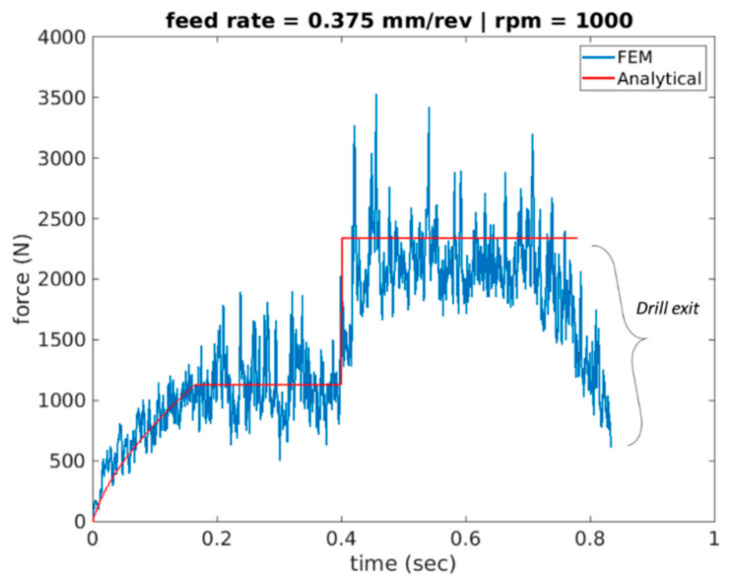
Force profile for scenario 4 of feed rate 0.375 mm/rev and 1000 spindle rpm (Analytical vs. FEA).

**Figure 16:**
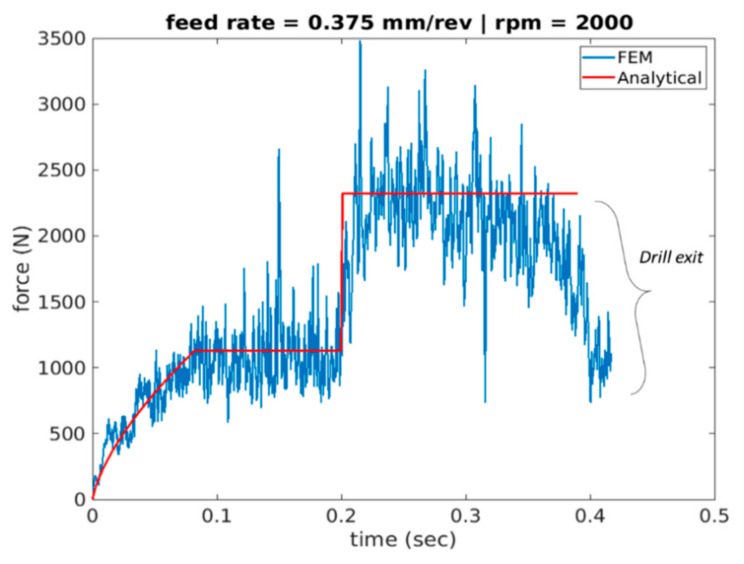
Force profile for scenario 5 of feed rate 0.375 mm/rev and 2000 spindle rpm (Analytical vs. FEA).

The associated bar charts for the first three simulation scenarios are shown in Figure 14. Relative error was found in the range of 11% for all scenarios, but only one scenario of stage II for 3000 rpm and 0.75 feed rate touching error around 25%. This profile in the force profile during entry was also observed in Figure 14, Figure 15 and Figure 16 that plot the cutting forces at a federate of 0.375 mm/rev and for cutting speeds of 1000 rpm, 2000 rpm and 3000 rpm respectively.

**Figure 17:**
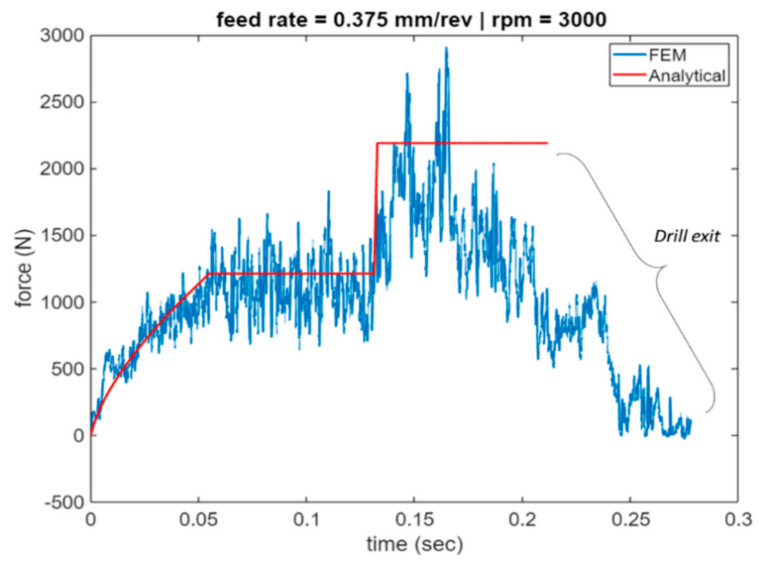
Force profile for scenario 6 of feed rate 0.375 mm/rev and 1000 spindle rpm (Analytical vs. FEA).

Figure 15, Figure 16 and Figure 17 plot the cutting force profiles of both FEA and analytical predictions simulation scenarios 4–6. More specifically, those are for the cutting speeds of 1000, 2000 and 3000 rpm, respectively, and a feed rate of 0.375 mm/rev. The analytical model is observed to be in line with the force entry profiles trends seen in literature [8]. 

More specifically, the force is seen to have a convex profile at entry as portrayed in stage I of all 6 drilling scenarios. It is worth noting here that the durations of each stage are calculated for each of the drilling scenarios based on the depth of the pilot hole, the depth of the workpiece, point angle k, feed rate and spindle speed. It has been observed that in stage III once the cutting force level has been reached during cutting it starts to decrease towards the hole exit. This decrease in thrust force is attributed to the thermal softening phenomenon. 

**Figure 18:**
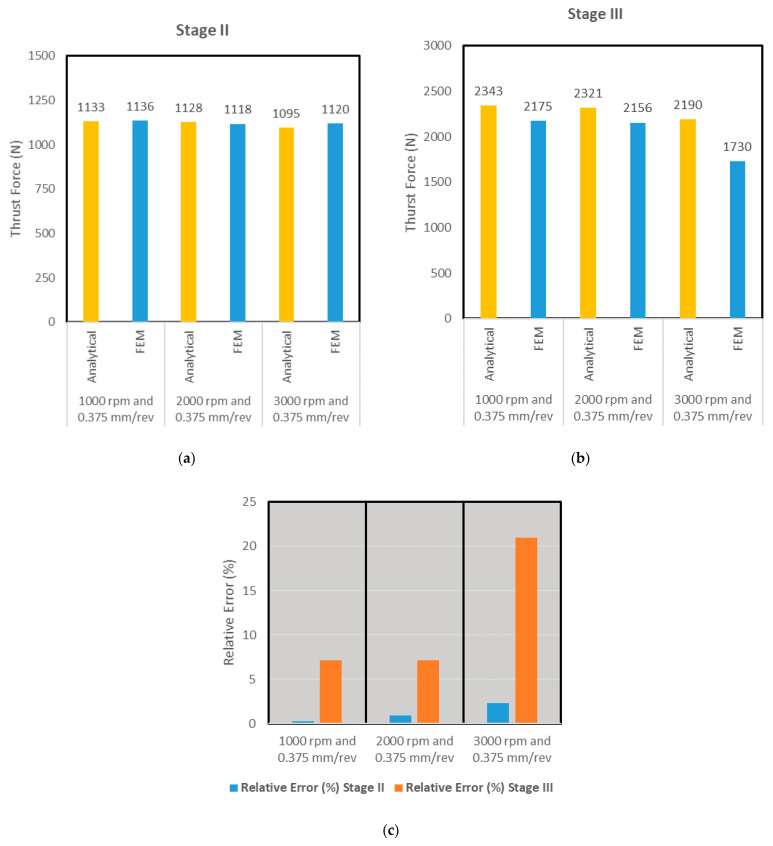
(**a**) Stage II: Average thrust forces in analytical and FEA approaches (**b**) Stage III: Average thrust forces in analytical and FEA approaches (**c**) Relative error % in both approaches.

Similar to Figure 14, the bar charts of Figure 18 reveal the average force comparison for stages II and III of scenarios 4–6 where the lower feed rate of 0.375 mm/rev is used. Relative error was found in the range of 7% for all scenarios, though only one scenario of stage III where the spindle speed was 3000 rpm and 0.375, the relative error between the two approaches revealed around 20% discrepancy. This was attributed to the lower magnitude of cutting force predicted using FEA due to the fact that thermal softening effects are more prevalent at high spindle speeds.

## 5. Conclusions

The following conclusions were drawn from the current study:

- The higher feed rate of 0.75 mm/rev provided more cutting temperature than lower feed level of 0.375 mm/rev. This was associated with the higher chip load at higher feed rate. Higher chip load can form higher chip root temperature at the tooltip under the cutting edge.
- Increasing the cutting speed resulted in an increase in the cutting temperature. The increase in temperature was associated with the fact that increasing cutting speed increases friction at the cutting zone.
- The cutting forces predicted by the FEA showed discrepancy when compared to those predicted by the analytical mode at the higher speed of 3000 rpm. This was attributed with the fact that the analytical model does not capture the material thermal softening phenomena, unlike the FEA model.
- The power law-based material model for Inconel 718 was developed in this work that captured the influence of strain hardening, strain rate sensitivity and thermal softening behavior. The model parameters can be utilized by other researchers to simulate the machining processes of similar nature.
- It was found that the thrust force at the entry profile of Inconel 718 was observed to follow a convex trend similar to what is found in the literature, even at high cutting velocities. It is because of the variation in the cutting pressure with respect to the cutting lip.
- While the computational power of computers has been advancing drastically, the study presented here revealed the computational time savings one can achieve by using the mechanistic analytical model to capture the force data in an Inconel drilling operation.
- The FEA-assisted numerical model accommodates a pilot hole in the current study making it unique as compared to other studies available in the literature. The presence of the pilot hole enabled us to capture the cutting force signature and cutting action of the chisel edge and cutting lip during the drilling operation. Overall, the analytical and FEA numerical models were found in good agreement with one another in predicting the cutting forces in drilling Inconel 718 material. Comparing the average forces of stage II and stage III of the two approaches revealed a discrepancy of 11% and 7% at most.

## Figures and Tables

**Figure 6:**
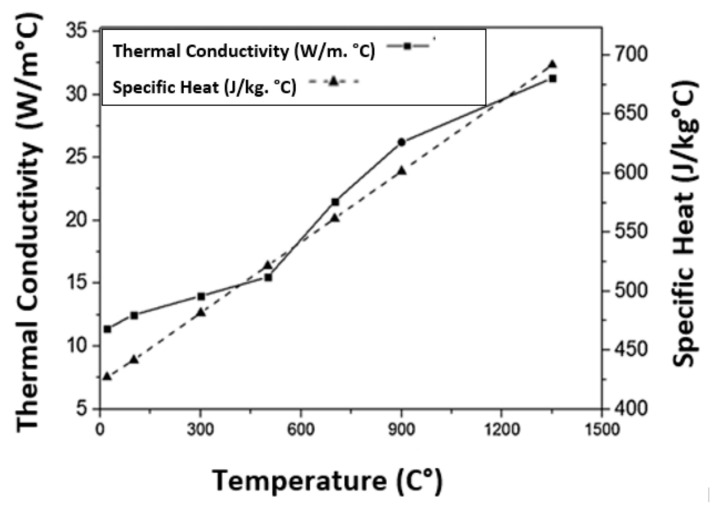
Thermal conductivity and heat capacity of Inconel 718 as a function of temperature (adopted from [30] with kind permission from Elsevier).

**Table 1:** Drill geometric parameters.

Symbol	Description	Value	Unit
ψ	chisel edge angle	120	degrees
k	(point angle)/2	135/2	degrees
$r_{p}$	pilot hole radius	1	mm
R	drill radius	3	mm
$d_{p}$	pilot hole depth	2	mm
$d_{w}$	work piece depth	5	mm
w	(web thickness)/2	0.5	mm

**Table 2:** Inconel 718 power law-based material parameters used in the FE model.

*σ*_0_ (MPa)	*n*	$\varepsilon_{o}^{p}$	*T_m_* (C°)	*T_cut_* (C°)	*m*1
1375	11.3	0.01	1420	700	100
***c*_0_**	***c*_1_**	***c*_2_**	***c*_3_**	***c*_4_**	***c*_5_**
1.004	−2 × 10^−3^	−1 × 10^−6^	5 × 10^−9^	−4 × 10^−12^	0

**Table 3:** Constants for the damage model parameters.

d_0_	d_1_	d_2_	d_3_	d_4_	d_5_
1.1404	8 × 10^−4^	3 × 10^−20^	0	0	0

**Table 4:** Drilling parameters for the six FEA scenarios.

Scenario No.	Feed Rate (mm/rev)	Spindle Speed (rpm)
1	0.75	1000
2	0.75	2000
3	0.75	3000
4	0.375	1000
5	0.375	2000
6	0.375	3000

## Data Availability

The data that support the findings of this study are available on request from the corresponding author, [W.A.S.].

## Abbreviations

$\alpha_{n}$	Chisel edge normal rake angle
$\beta_{o}$	Clearance angle
$C_{1}$	Constant dependent on drill geometry & machining conditions
$C_{2}$	Integration constant
ψ	Chisel edge angle
$f_{rev}$	Feed rate in mm/rev
$f_{s}$	Feed rate in mm/sec
i	Inclination angle
k	(Point angle)/2
$K_{n}$	Norma specific cutting pressure
$\tau_{y}$	Yield shear stress
r	Radial distance along cutting lips of the drill
$r_{p}$	Pilot hole radius
ρ	Normalized radial coordinate
R	Drill radius
$R_{a}$	Indentation zone radius
t	time
$t_{c}$	Uncut chip thickness
$F_{cl}$	Cutting lips force
$F_{ind}$	Indentation zone force
$V_{c}$	Cutting velocity
w	(Web thickness)/2
*g* ($\varepsilon^{p}$)	Strain hardening function
*θ*(*T*)	Thermal softening function
*τ* ($\dot{\varepsilon }$)	Rate sensitivity function
*σo*	Initial yield stress is, is referred as
$\varepsilon^{p}$	Plastic strain
$\varepsilon_{o}^{p}$	Reference plastic strain
1/ *n*	Strain hardening power
*T*	Temperature during the test
*T_m_*	Melting temperature
*T_cut_*	Cut-off temperature
$\dot{\varepsilon }$	Plastic strain rate
${\dot{\varepsilon }}_{o}$	Reference plastic strain rate
*m*_1_	Strain rate sensitivity
*F_s_*	Sliding force
*μ*	Coefficient of friction
*σ_n_*	Normal force
*D*	Damage function
